# Dual-Ion Co-Regulation System Enabling High-Performance Electrochemical Artificial Yarn Muscles with Energy-Free Catch States

**DOI:** 10.1007/s40820-023-01133-2

**Published:** 2023-06-29

**Authors:** Ming Ren, Lizhong Dong, Xiaobo Wang, Yuxin Li, Yueran Zhao, Bo Cui, Guang Yang, Wei Li, Xiaojie Yuan, Tao Zhou, Panpan Xu, Xiaona Wang, Jiangtao Di, Qingwen Li

**Affiliations:** 1 Key Laboratory of Multifunctional Nanomaterials and Smart Systems, Advanced Materials Division, Suzhou Institute of Nano-Tech and Nano-Bionics, Chinese Academy of Sciences, Suzhou, 215123 People’s Republic of China; 2 School of Nano-Technology and Nano-Bionics, University of Science and Technology of China, Hefei, 230026 People’s Republic of China; 3 Division of Nanomaterials and Jiangxi Key Lab of Carbonene Materials, Jiangxi Institute of Nanotechnology, Nanchang, 330200 People’s Republic of China

**Keywords:** Artificial muscles, Carbon nanotube yarns, Electrochemical actuators, Catch state, Dual-ion co-regulation

## Abstract

Artificial yarn muscles show great potential in applications requiring low-energy consumption while maintaining high performance. However, conventional designs have been limited by weak ion-yarn muscle interactions and inefficient “rocking-chair” ion migration. To address these limitations, we present an electrochemical artificial yarn muscle design driven by a dual-ion co-regulation system. By utilizing two reaction channels, this system shortens ion migration pathways, leading to faster and more efficient actuation. During the charging/discharging process, ${\text{PF}}_{6}^{ - }$ ions react with carbon nanotube yarn, while Li^+^ ions react with an Al foil. The intercalation reaction between ${\text{PF}}_{6}^{ - }$ and collapsed carbon nanotubes allows the yarn muscle to achieve an energy-free high-tension catch state. The dual-ion coordinated yarn muscles exhibit superior contractile stroke, maximum contractile rate, and maximum power densities, exceeding those of “rocking-chair” type ion migration yarn muscles. The dual-ion co-regulation system enhances the ion migration rate during actuation, resulting in improved performance. Moreover, the yarn muscles can withstand high levels of isometric stress, displaying a stress of 61 times that of skeletal muscles and 8 times that of “rocking-chair” type yarn muscles at higher frequencies. This technology holds significant potential for various applications, including prosthetics and robotics.

**Supplementary Information:**

The online version contains supplementary material available at 10.1007/s40820-023-01133-2.

## Highlights

- The dual-ion co-regulation system shortens ion migration pathways, which endow the yarn muscle with high contractile stroke (34.7%) and contractile rate (9.4% s^−1^), more than twice that of the “rocking-chair” -type ion migration yarn muscles.
- The yarn muscle shows high isometric stress of 18.4 MPa (61 times that of skeletal muscles) and 8 times the isometric stress of the “rocking-chair” -type yarn muscles at a higher frequency.
- The intercalation reaction between ${\text{PF}}_{6}^{ - }$and collapsed carbon nanotubes allows the yarn muscle to achieve an energy-free high-tension catch state

**Supplementary Information:**

The online version contains supplementary material available at 10.1007/s40820-023-01133-2.

## Graphical abstract

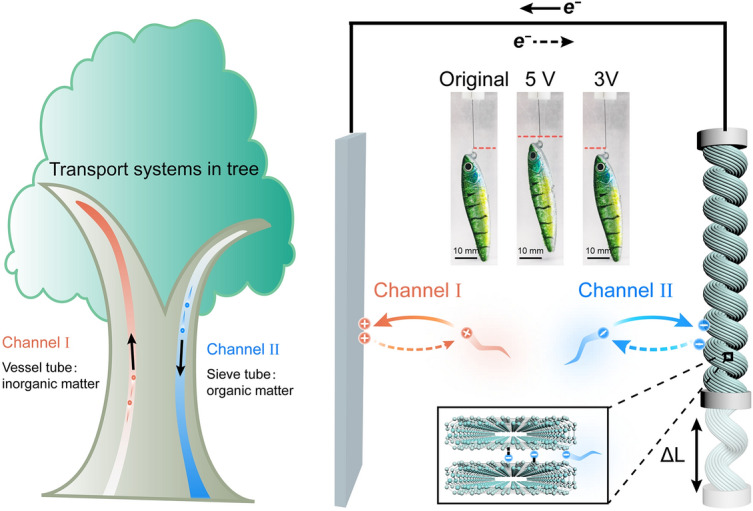

## Introduction

Inspired by skeletal muscle in nature, artificial muscle yarns that can reversibly contract or rotate under external stimulation (heat, electrochemical ion injection, or solvent) have been developed and are considered promising candidates for application in the field of soft robots [1-5]. The reversible shape changes of artificial muscle yarns are achieved by changing the molecular chain arrangement inside the yarns or injecting the guest within the yarns [6, 7]. Baughman et al. discovered that the shape changes could be amplified by twisting artificial muscles into coiled structures [8, 9], which expands the range of materials of artificial muscle yarns, including carbon nanotube (CNT) yarns [10, 11], polymer fibers [8, 12], shape memory alloy wires [13], natural fibers [14], etc.

Electrochemical artificial muscle yarns based on double-layer ions injection have attracted wide interest recently due to their low driven voltage, negligible thermal effect, and better controllability [15-19]. Foroughi et al. [20] reported a twisted CNT yarn that contracted about 1% of its length when charged at −5.0 V (vs. Ag/Ag^+^ reference) in an organic electrolyte. To enhance contractile stroke, the CNT yarn was fully twisted and the contractile stroke of the CNT yarn increased to 16.5% in tetrabutylammonium hexafluorophosphate acetonitrile electrolyte. On the other hand, the contractile stroke can be enhanced by fast ion transport. Qiao et al. [21] reported an electrochemical CNT/graphene hybrid yarn muscles that generated 8.1% contractile stroke in aqueous electrolytes, which was two times that of neat CNT yarn. That is due to the biscrolled structure formed within the hybrid, which provides rapid ion transport channels. Mu et al. [15] demonstrated the CNT-sheathed nylon 6 fiber muscle generates contractile stroke of 14.3% and 1.98 W g^−1^ of average contractile power that was 9.0 times that of the alternative electrochemical muscles. The sheath-run structure shortens the pathway for ion migration in the electrochemical processes. Due to the weak interaction strength between ions and the muscle yarns [22], the electrochemical yarn muscles actuated by double-layer charging cannot maintain the generated displacement without continuous stimulation and energy consumption [23, 24].

Enhancing the interaction between ions and muscle yarns by introducing intercalation reactions is an ideal strategy to address this challenge. We have previously developed a CNT yarn muscle driven by aluminum-ion insertion, which deployed a mechanism of electrochemically reversible insertion reaction between tetrachloroaluminate ions and collapsed CNTs in the yarn [25]. This insertion reaction endowed the yarn muscle to hold nearly 100% of the achieved contraction states after being powered off (catch state). However, the “rocking-chair”-type migration of tetrachloroaluminate ions increased the charge transport pathways, which lead to low contractile stroke, and slow actuation responses. For example, during the charging process, the AlCl$_{4}^{ - }$migrated to the cathode and reacted with CNTs as follows: ${\text{C}}_{{\text{n}}} + {\text{AlCl}}_{{4}}^{ - } \to {\text{C}}_{{\text{n}}} \left[ {{\text{AlCl}}_{{4}} } \right] + {\text{e}}^{ - }$. While AlCl$_{4}^{ - }$ migrated to the anode and reacted with the Al foil as follows: ${\text{7AlCl}}_{{4}}^{ - } + {\text{Al}} \to {\text{4Al}}_{{2}} {\text{Cl}}_{7}^{{ - }{}} + {\text{3e}}^{ - }$. This resulted that a stroke was only 15%, and the contractile rate was only 5% s^−1^ [25]. Therefore, shortening the pathways of ion migration provides a way to improve the actuation performance of artificial yarn muscles with catch states.

Herein, we reported a dual-ion coordinated electrochemical artificial yarn muscle that can generate large contractile stroke and contractile rate while maintaining a catch state. The dual-ion co-regulation system shortens ion migration pathways by changing the “rocking-chair”-type migration into two reaction channels. During the charging process, the anion and cation undergo an intercalation reaction and alloying reaction with the cathode and anode, respectively. During the discharging process, both anion and cation are released back into the electrolyte. This reaction mechanism enhances the ion migration rate during actuating, which allows the yarn muscle to show a contractile stroke of 34.7%, a maximum contractile rate of 9.4% s^−1^, and a maximum power density of 0.37 W g^−1^, more than twice that of the “rocking-chair”-type ion migration yarn muscles. This strategy also results in dual-ion coordinated yarn muscle (DIYM) generating 8 times the isometric stress of the “rocking-chair”-type yarn muscle (RCYM) at a higher frequency.

## Experimental Section

### Materials

Ethanol was purchased from Sinopharm Chemical Reagent Co., Ltd. Ferrocene and thiophene were purchased from Aladdin Chemical Reagent Co., Ltd. 1 mol L^−1^ lithium hexafluorophosphate (LiPF_6_) in propylene carbonate (PC) with a 5 vol% fluoroethylene carbonate (FEC) (if not specified, the concentration of LiPF_6_ is 1 mol L^−1^ and the volume ratio of FEC is 5% in this paper) and AlCl_3_/1-ethyl-3-methylimidazolium ([EMIm] Cl) (mole ratio of AlCl_3_ to [EMIm]Cl was 1.3) were purchased from Suzhou Duoduo Chemical Technology Co., Ltd. Aluminum foil with 0.5 mm thickness was purchased commercially. All reagents are purchased and used directly without further purification.

### Preparation of CNT Coiled Yarns

The carbon nanotube yarns utilized here were fabricated by a floating catalyst chemical vapor deposition (FCCVD) method [26]. Briefly, the mixed solution with the ethanol (carbon source), ferrocene (catalyst, 1–3 wt%), thiophene (catalytic aid, 2–5 wt%), and deionized water (etchant, 2–5 wt%) was injected into the reaction furnace with the temperature for the growth reaction of 1100–1300 °C. A continuous CNT aerogel was spontaneously formed in the carrier gas composed of H_2_/Ar mixed gas and was drawn from the end of the furnace. After densifying the CNT aerogel through a water tank, narrow CNT ribbons were obtained. One end of the CNT ribbon was suspended vertically on a motor, and the other end was suspended with a load of 2 g. The fully coiled CNT yarn was formed by inserting a twist density of 25,000 turns m^−1^ into the ribbon. And the coiled CNT yarn was utilized as the artificial muscle.

### Structure and Spectroscopy Characterizations

The morphologies of CNT yarn were characterized by a field emission scanning electron microscope (SEM, FEI Quanta FEG 250) operated at 10 kV. The cross section of CNT yarn was obtained by radially sectioning the CNT to a depth of 10 μm from the surface using a focused ion beam (FIB) operating at 30 kV (Thermo Fisher Helos 5 UX). The field emission transmission electron microscope (TEM) image of the cross section was obtained using Talos F200X (Thermo Fisher). The Raman spectroscopy was performed by using a HeNe laser with a wavelength of 532 nm (Renishaw in Via Qantor). The yarns with different states were obtained by charging/discharging during the cyclic voltammetry (CV) scan between 3 and 5 V at 10 mV s^−1^. After the reaction, the yarns were removed from the glovebox to characterize the Raman spectroscopy.

### Electrochemical and Actuation Measurements

All electrochemical measurements were performed by CHI 660E electrochemical workstation from CH Instruments, Inc. The dual-ion co-regulation system was composed of two electrodes. The coiled CNT yarn was used as a cathode electrode, the aluminum foil was used as an anode, and the LiPF_6_ in PC with FEC was used as an electrolyte. The “rocking-chair”-type system was composed of the two electrodes of the coiled CNT yarn and the aluminum foil, and the AlCl_3_/[EMIm]Cl (mole ratio of AlCl_3_ to [EMIm]Cl was 1.3) was used as an electrolyte. All the electrochemical measurements were performed in the glovebox. The electrical stimulation was applied by using an electrochemical workstation. The contractile displacements (*L*) of the artificial muscle were recorded using a contactless electromagnetic sensor. The generated isometric force (*F*) of the yarn muscle was recorded by a load cell (JZ-101, XINHANG). The actuation performance was evaluated at different parameters of electrical stimulations and applied loads.

### Calculation

The contractile stroke (*ε*) of the artificial muscle was calculated by Eq. (1),

1 $${ }\varepsilon \left( \% \right) = \frac{{{\Delta }L}}{L}{ } \times { 1}00$$

where *L* is the initial length of the artificial muscles, and *ΔL* is the contractile displacement of the artificial muscle under the voltage applied.

The contractile rate (*ν*) of the artificial muscle was calculated by Eq. (2)

2 $$\nu (\% {\text{ s}}^{{ - {1}}} ) = \frac{{{\text{d}}\varepsilon }}{{{\text{dt}}}}$$

where *ε* is the contractile stroke, and *t* is the time.

The contractile work density (*W*) was calculated by Eq. (3),

3 $$W({\text{J g}}^{{ - {1}}} ) = \frac{Mgh}{m}$$

where *M* is the weight of the applied load, *g* is the gravitational acceleration, *h* is the contractile displacement, and *m* is the weight of artificial muscle.

The catch index (*i*) was calculated by Eq. (4),

4 $$i\left( \% \right) = \frac{{\varepsilon_{t} }}{{\varepsilon_{0} }} \times { 1}00$$

where *ε*_*t*_ is the real-time stroke after powering off, and *ε*_*0*_ is the stroke achieved just before powering off.

The generated isometric stress (*σ*) was calculated by Eq. (5),

5 $$\sigma \left( {MPa} \right) = \frac{F}{S}$$

where *F* is the generated isometric force, and *S* is the cross-sectional area.

The effect conversion efficiency (*η*) was calculated by Eq. (6),

6 $$\eta \, \left( \% \right) = \frac{{W_{{{\text{output}}}} }}{{W_{{{\text{input}}}} }}$$

where *η* is the energy conversion efficiency, *W*_output_ is the net output mechanical energy, and *W*_input_ is the net input electrical energy.

## Results and Discussion

### Preparation and Characterization of DIYM

**Fig. 1:**
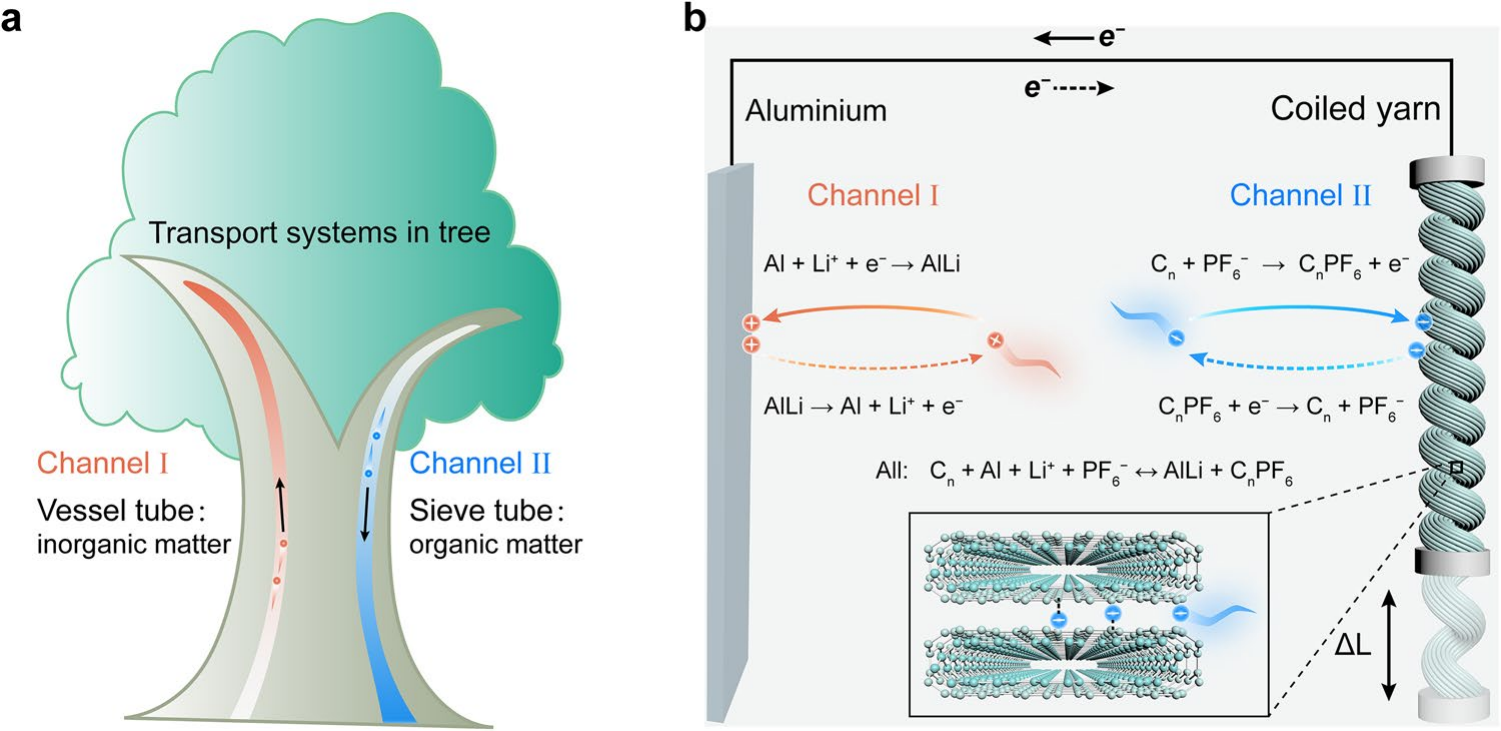
Schematic illustration of the DIYM. **a** Transport system of matters in the tree. **b** Structural and reaction equations of the actuation system consisted of an Al foil as the anode, a CNT coiled yarn as the cathode, and LiPF_6_ in PC with FEC as the electrolyte. The insert shows the insertion of ${\text{PF}}_{6}^{ - }$ into collapsed CNTs

The natural trees show an efficient mass transfer process using the two channels of the vessel tube and sieve tube to transport inorganic and organic matter, respectively (Fig. 1a) [27], which inspires the design of the DIYM. Two reaction channels that replace the single channel of the “rocking-chair” are built for ions quickly migrating [28]. The yarn muscle work system for actuation is composed of two electrodes with a coiled CNT yarn muscle as the working electrode and an aluminum foil as the counter electrode, while LiPF_6_ in PC with FEC is used as the electrolyte (Fig. 1b). The reaction equations involved in the charging and discharging process are shown in Fig. 1b [29]. During the charging process, the ${\text{PF}}_{6}^{ - }$ anion intercalates into the collapsed CNTs and forms intercalation compounds (insert in Fig. 1b). The stronger interaction between ${\text{PF}}_{6}^{ - }$ and collapsed CNTs generated by the intercalation reaction enables the yarn muscle to maintain a catch state. Meanwhile, the Li^+^ reacts with an aluminum electrode to form LiAl alloy. During the discharging process, the ${\text{PF}}_{6}^{ - }$ de-intercalates from the collapsed CNTs in the yarn, while the Li^**+**^ de-alloy from LiAl, and both Li^+^ and ${\text{PF}}_{6}^{ - }$ are released back from the electrodes into the electrolyte [30]. The intercalation of ${\text{PF}}_{6}^{ - }$ into coiled CNT yarn can be described as follow [31]: ${\text{C}}_{{\text{n}}} \, +  \, \, \text{PF}_{6}^{ - } \leftrightarrow \,{\text{C}}_{{\text{n}}} {\text{PF}}_{{6}} \, + \,{\text{e}}^{ - }$, where the C_n_PF_6_ is the intercalation compound, and *n* is the molar ratio of carbon atoms to ${\text{PF}}_{6}^{ - }$ in the CNT yarn. And the reaction of the Li^+^ alloying with aluminum Al + Li^+^ + e^−^ ↔ LiAl occurs correspondingly [32].

**Fig. 2:**
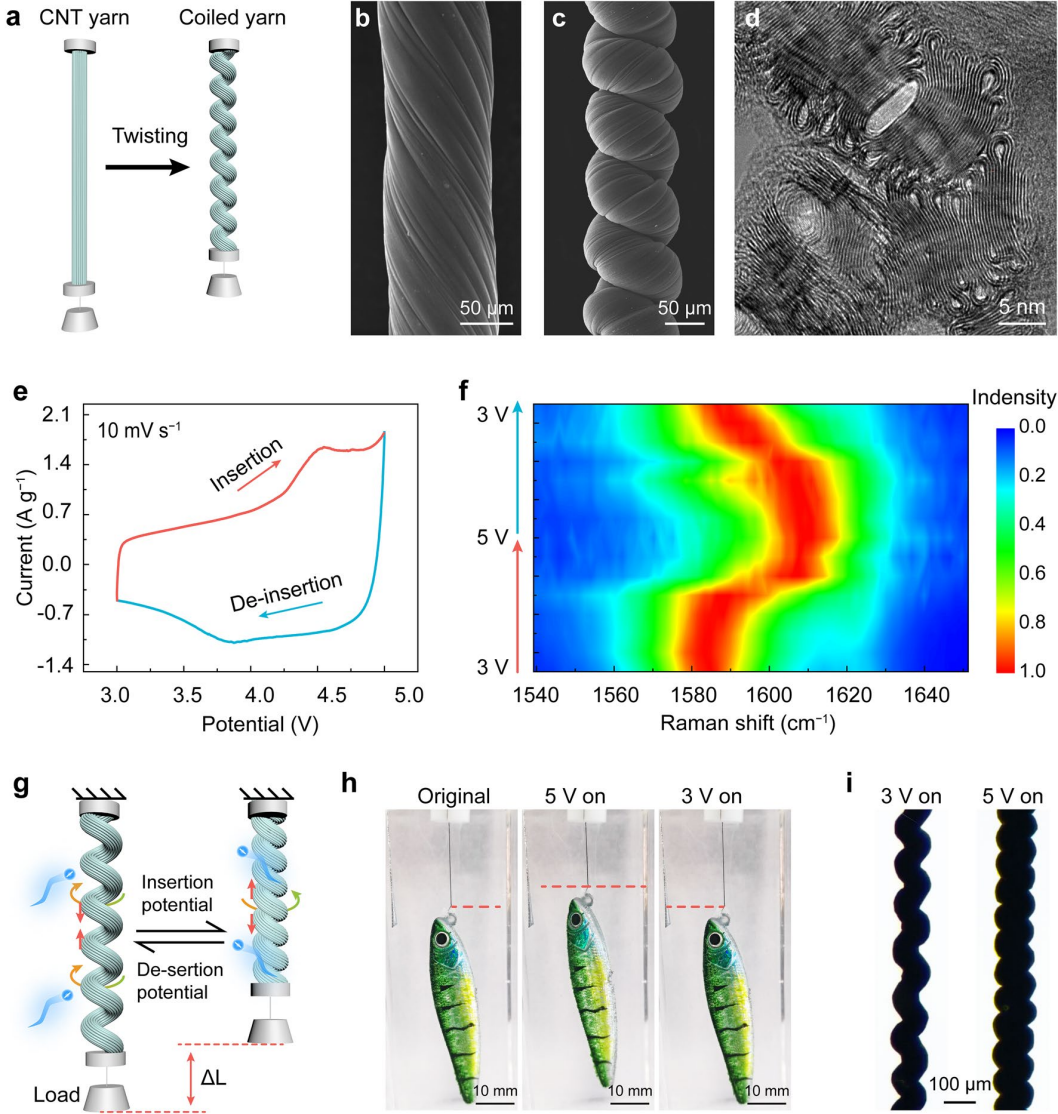
Structure and spectroscopy characterizations of the DIYM. **a** Schematic illustration of the twisting process. **b** SEM image of pristine twisted CNT yarn. **c** The SEM image of fully coiled CNT yarn. **d** TEM image of the cross-section of the coiled CNT yarn that shows the collapsed CNTs in the yarn. **e** CV curve of the yarn muscle that was carried out at 10 mV s^−1^ between 3 and 5 V versus Al foil. **f** Raman spectroscopy of coiled CNT yarn with different charging/discharging states. **g** Contraction mechanism of the electrochemical artificial muscle yarns. **h** Optical images of the yarn muscle as reversibly lifting a model fish with a 4-gram weight. **i** High-magnification images of the yarn muscle in (**h**)

The CNTs utilized for preparing artificial muscles were fabricated by a floating catalyst chemical vapor deposition method (see the Experimental Section for details) [26]. In this method, the carbon source was injected into a high-temperature reaction furnace (about 1300 °C), and a continuous CNT aerogel sheet was carried out by the carrier gas. The pristine CNT ribbon was obtained by passing the aerogel sheet through a water bath. The CNT ribbon has a width of about 830 μm and the thickness of the ribbon is about 18 μm (Fig. S1). The line resistance of the CNT ribbon is about 10 Ω cm^−1^(Fig. S2). And the failure strain is 10.8%, and the breaking strength is 104.5 MPa (Fig. S3). After the counterclockwise twisting process, the coiled CNT yarn was formed (Fig. 2a). SEM image in Fig. 2b shows the twisted yarn with a diameter of 103 μm and a bias angle of 35.1°. Continuing over twisting with a twist density of about 25,000 turns m^−1^, the yarn was fully coiled with a reduced fiber diameter of 73 μm (Fig. 2c), which was utilized as the artificial muscle. The TEM image of the cross-section of coiled yarn shows that the CNTs with about 2–3 graphitic walls (accounted for 81%) collapsed with the “dog bone” shape in the yarn (Fig. 2d) [33]. The interlayer spacing of the collapsed stacked structure (about 0.36 nm) similar to that of graphite (002, 0.34 nm) provides reaction sites for ion intercalation, which is not available in conventional tubular CNTs [34]. The ion intercalation reaction enhances the interaction between ions and CNTs compared with the electric double-layer adsorption mechanism, which provides artificial muscle yarns a catch state.

As presented in Fig. 2e, the CV curve of the coiled CNT yarn was performed with a scan potential range from 3 to 5 V at a scan rate of 10 mV s^−1^. The results show that the oxidation peaks appear around 4.5 V during the charging process, which was associated with the insertion of abundant ${\text{PF}}_{6}^{ - }$ into the collapsed CNTs forming the intercalation compound C_n_[PF_6_]. At the potential below about 4.2 V, the current was relatively small, in which the ion adsorption was dominated. During the discharging process, the reduction peak occurred at around 3.9 V due to the releasing back of ${\text{PF}}_{6}^{ - }$ from collapsed CNTs. The reduction peak was asymmetrical with the oxidation peak and shifted, which may be due to the kinetic differences between the intercalation and re-intercalation process, and this shifted overpotential also confirmed the strong interaction between ions and collapsed carbon nanotubes.

To further understand the ${\text{PF}}_{6}^{ - }$ intercalation/re-intercalation during the charging/discharging process, ex situ Raman spectroscopy was performed (Fig. 2f). The coiled CNT yarns were charged and discharged to different states. When the yarn muscle was charged from 3 to 4.2 V, the G peak blue-shifted slightly from 1585 to 1590 cm^−1^, which was due to the charge transfer between ${\text{PF}}_{6}^{ - }$ and CNTs during electrochemical double-layer adsorption process (Figs. S4 and S5). And a new peak was split from the G peak and overlapped with the D′ peak (at 1622 cm^−1^) with the potential increased to 4.5 V [35], which was attributed to the rearrangement of positive charges on graphite boundary layers [36]. As the coiled CNT yarn was charged to 5 V, the split new peak and the G peak after splitting blue-shifted to the maximum of 1612 and 1603 cm^−1^, respectively. Conversely, the two peaks gradually overlapped into one peak for the G band at 1588 cm^−1^ during the potential back to 3 V. The splitting and reversible shift of the G peak suggest the intercalation/re-intercalation of ${\text{PF}}_{6}^{ - }$ into/from the collapsed CNT bundle during the charging/discharging process.

The ion intercalation-caused contraction mechanism of artificial muscle yarns is shown in Fig. 2g. Before the test, a pretension (load) needs to be applied to the artificial muscle yarn to separate the adjacent coils for contraction. When intercalation potential was applied to the artificial muscles, the Li^+^ ion undergo an alloying reaction with Al foil while the ${\text{PF}}_{6}^{ - }$ ions intercalated into the artificial muscle [29]. The blue shift and splitting of the G peak in Raman spectroscopy confirm the ion intercalation of ${\text{PF}}_{6}^{ - }$ into the collapsed CNT bundle. The intercalated ions caused the radial volume expansion of artificial muscle yarn under the applied intercalation potential, which generated the untwisting torque and pulled the coils together, and the axial contraction of the coiled CNT yarn was generated [8, 9]. When the de-intercalation potential was applied, the Li^+^ ion de-alloy from LiAl alloy while the${\text{PF}}_{6}^{ - }$ ions de-intercalated from the coiled CNT yarns [30], and the coils were separated from each other by tension resulting in the artificial muscle yarn returning to its original length. To fully intercalate/de-intercalate ions into/from collapsed CNTs to produce greater actuation performance, the stimulation potential range of the electrochemical artificial muscle is set at 3 to 5 V. The optical images in Fig. 2h, i visually show the contraction and return processes. An artificial muscle yarn with a 21-mm length lifted a fish with a 4-gram mass by 5.5 mm under the applied potential of 5 V. When the potential turned to 3 V, the contraction of the artificial muscle yarn disappeared (Movie S1). The high-magnification images exhibit that the diameter of the yarn muscle increased and coils were close together when the potential was switched from 3 to 5 V. This contraction mechanism enables the states of artificial muscle yarn to be regulated by switching the potential between the intercalation and the de-intercalation.

### Contractile Actuation Performance of DIYM

**Fig. 3:**
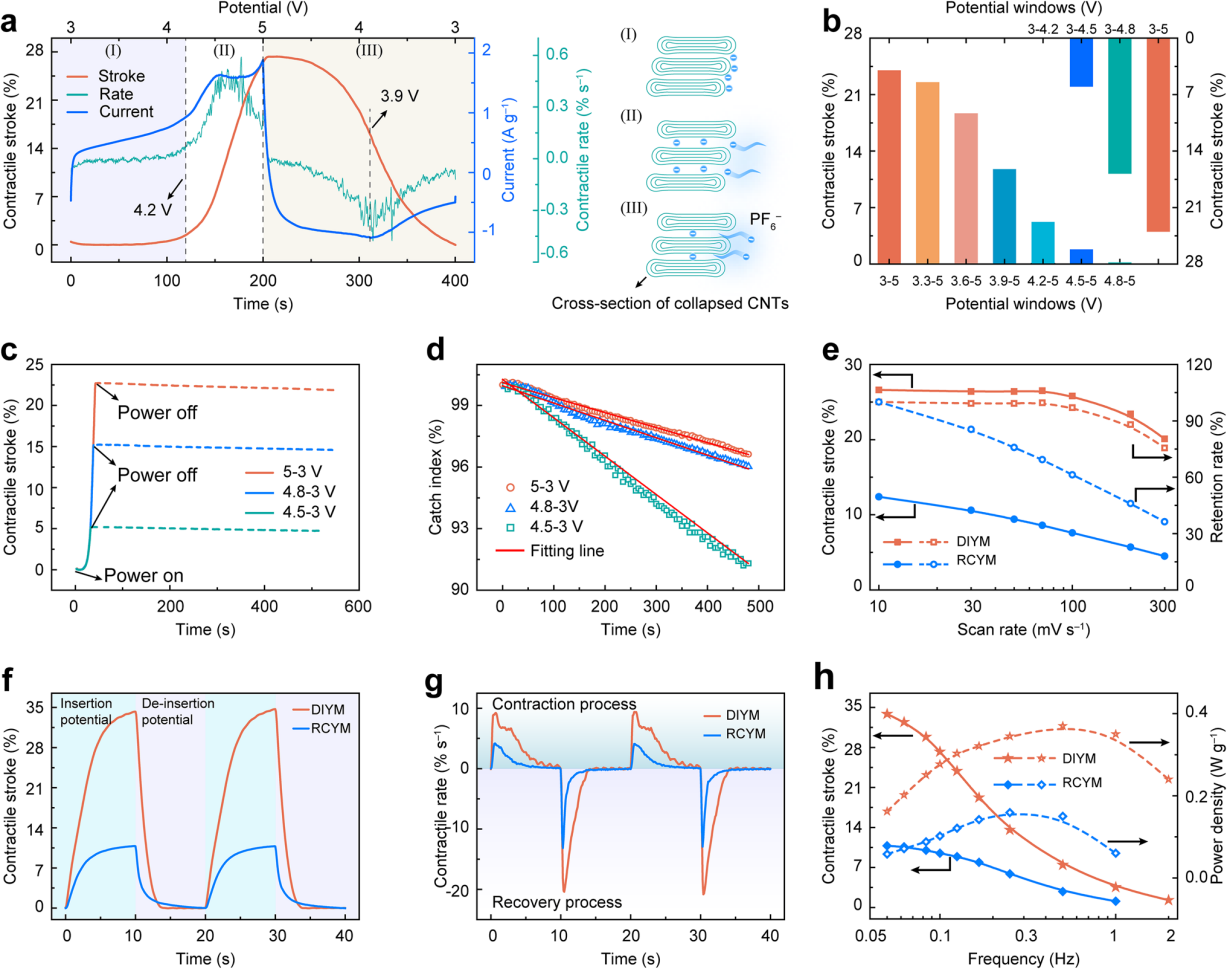
The contractile actuation performance of DIYM. **a** Contractile stroke, contractile rate, and current versus time during a full CV scan between 3 and 5 V at 10 mV s^−1^ for a coiled yarn bearing 10 MPa applied stress. The corresponding potential profile is shown on the top x-axis. The right shows the cross-section of collapsed CNTs and the distribution of ${\text{PF}}_{6}^{ - }$ during stages I, II, and III, respectively. **b** The contractile stroke of the yarn muscle at different voltage windows at a scan rate of 50 mV s^−1^ under a 10 MPa applied stress. **c** The contractile stroke versus time, during potential charging from 3 V at a scan rate of 50 mV s^−1^ to target potential (solid line) and then powered off for 500 s (dash line). The tension applied to the muscle is 10 MPa. **d** The catch index of the yarn muscle that was charged from 3 to 4.5, 4.8, and 5 V, respectively, at 50 mV s^−1^ under the tension loads of 10 MPa. **e** The comparison of the contractile stroke and its retention of the DIYM and RCYM at different scan rates from 10 to 300 mV s^−1^ and the applied tension loads is 10 MPa. **f** The contractile stroke curves under a frequency of 0.05 Hz when a 3 to 5 V square wave for the DIYM and 0.2 to 2.2 V square ware for the RCYM with a 50% duty cycle and tension stress of 10 MPa were applied. **g** The contractile rates during the contraction process in (**g**) were calculated by deriving the time from the contractile stroke. **h** The contractile strokes and power densities during the contraction process of both the DIYM and RCYM at different frequencies

The contractile actuation performance of DIYM was investigated. The schematic illustration of the apparatus for contractile actuation performance characterization is shown in Fig. S6. As presented in Fig. 3a, the time dependence of contractile stroke and the current was characterized during a full cycle of CV scan between 3 and 5 V at 10 mV s^−1^ for a coiled CNT yarn under the applied load of 10 MPa. The curve of contractile stroke could be roughly divided into three stages according to the values of contraction and contractile rate. In the first stage which corresponds to the potential increasing from 3 to 4.2 V, the value of contractile stroke and contractile rate were negligible, while the current remained positive and gradually increased. The charges induced by the increased current were adsorbed from the electrolyte onto the surface of the CNT bundles, which produced a small effective volume expansion. In the second stage, the artificial muscle yarn rapidly contracted from 1.5% to 27% and the contractile rate increased from 0.07% to 0.45% s^−1^. This suggests that massive amounts of ${\text{PF}}_{6}^{ - }$ were intercalated into the collapsed CNTs. The third stage corresponds to the discharge process where the potential decreased from 5 to 3 V. The contractile stroke gradually decreased to 0% due to the de-intercalation reaction $({\text{C}}_{{\text{n}}} \left[ {{\text{PF}}_{{6}} } \right] + {\text{e}}^{ - } \to {\text{C}}_{{\text{n}}} + {\text{PF}}_{6}^{ - } )$ . The contractile rate became negative (the return process of artificial muscle) with the value of current becoming negative and reached the maximum of −0.33% s^−1^ at around about 3.9 V (reduction peak) and then gradually decreased to 0. The curves of the contractile stroke and rate show the effect of intercalation reaction on contraction actuation performance.

To further investigate the potential dependence of the contractile stroke, we measured the contractile strokes of the yarn muscle using a CV scan ranging from different onset potentials to 5 V (Fig. S7) and from 3 V to different end potentials (Fig. S8) at 50 mV s^−1^ and an applied load of 10 MPa. The different CV curves show redox peaks occur simultaneously, and when the onset potential was above 3.9 V or the end potential was below 4.5 V, the oxidation peak and the reduction peak disappeared. The contractile stroke of the yarn muscle under different CV scan potentials was presented in Fig. 3b, where the bottom X-axis showed the results of different onset potentials, and the top X-axis showed the result of different end potentials. When the onset potential was above 3.9 V, the contractile strokes were significantly reduced due to the relatively slow reaction kinetics of the de-intercalation process $\left( {{\text{C}}_{{\text{n}}} \left[ {{\text{PF}}_{{6}} } \right] + {\text{e}}^{ - } \to {\text{C}}_{{\text{n}}} + {\text{PF}}_{6}^{ - } } \right)$ and slow release of ${\text{PF}}_{6}^{ - }$ from the coiled CNT yarn reducing the reversible contraction of artificial muscle. On the other hand, the lack of accumulated ions on the surface of CNT bundles by adsorption in the relatively low potential ranges could also hinder the contraction generated by intercalation. For the result of different end potentials, the contractile stroke of the yarn muscle was nearly negligible despite the existence of ion injection when the end potential was below 4.2 V. This was due to the injected ${\text{PF}}_{6}^{ - }$ from the electrolyte being adsorbed on the surface of the CNT bundles and generated small volume expansion. This confirms that intercalation and de-intercalation reactions determine the contraction actuation performance of artificial muscle yarns.

The intercalation reaction between ${\text{PF}}_{6}^{ - }$ ions and collapsed CNTs generates the intercalation compound C_n_[PF_6_], which enhances the interaction between ions and CNT yarns. The catch state of the artificial muscle yarns was characterized by recording the contractile stroke versus time after stopping voltage stimulation. The artificial muscle yarn was charged from 3 to 4.5, 4.8, and 5 V at a scan rate of 50 mV s^−1^, respectively, and then the power was turned off. The curves that are shown in Fig. 3c where the solid line represents the contractile stroke during charging and the dashed line represents that after the power was turned off. The three curves coincide during charging and remain relatively stable after the power was turned off for 500 s even when a load of 19,000 times the muscle weight was applied. The catch index of the yarn muscle after power-off was further analyzed in Fig. 3d. The curve of the catch index exhibited basically linear decay over time and the curve slope decreased with an increase of the end potential, which was due to more intercalation compounds generated at higher end potential. After the power was off for 500 s, the catch indices of yarn muscle with the end potentials of 4.5, 4.8, and 5 V were 91.2%, 95.8%, and 96.4%, respectively, and the decay rates of the catch index were 0.02%, 0.008%, and 0.007% s^−1^, respectively, as shown in Fig. S9. These results confirmed the intercalation reaction enhanced the interaction between ions and CNT yarns allowing the yarn muscle to catch state.

We then investigated the dependence of contractile stroke at different CV scan rates. The curve of contractile stroke versus time at different scan rates during CV measurements showed the stroke maintained stability even the scan rate increased to 100 mV s^−1^ (Fig. S10). In contrast, the contractile stroke of RCYM decreased with the scan rates increasing (Fig. S11). The comparison of the contractile stroke and its retention of the two types of artificial muscles under different scan rates are shown in Fig. 3e. The contractile stroke of the DIYM decreased from 26.6% to 20.1%, and the retention of contractile stroke was 76% when the scan rate increased from 10 to 300 mV s^−1^, while at the same scan rates as above, the contractile stroke of the RCYM was significantly decreased from 12.4% to 4.5%, and the retention of contractile stroke was only 36%. The higher contractile stroke and its retention at a high scan rate of the DIYM were due to the dual-ion co-regulation system shortened ion migration pathways and enhanced ion migration rate. The maximum contraction work and energy conversion efficiency of DIYM at different scan rates were about 1.1 J g^−1^ and 1.3%, respectively, which were 1.7 and 1.2 times that of the RCYM (Fig. S12).

The actuation performance was further discussed by applying a square-wave potential. The curves of contractile stroke of the two types of yarn muscles are shown in Fig. 3f when a square wave at 0.05 Hz with a 50% duty cycle (if not specified, the square wave voltage with a 50% duty cycle is used in this paper) and a pretension of 10 MPa was applied. The contractile stroke of DIYM gradually increased to 34.7% when the potential of 5 V was applied for 10 s, and the yarn muscles returned to the original length when the applied voltage was switched to 3 V. The maximum contractile stroke of the RCYM was only 10.8% when a 0.05 Hz, 0.2–2.2 V square wave was applied. The contractile stroke value of DIYM is higher than that of skeletal muscles (20%) [37] and most of other reported electrochemical artificial muscles (Fig. S13), such as SWNT sheet (0.1%) [38], CNT-rGO yarn (8.1%) [21], CNT@PVDF yarn (10.4%) [18], Nylon/CNT (14.3%) [15], Two-ply CNT yarn (16.5%), [10] and CNT yarn (18.1%) [25]. The contractile rates during the contraction process in Fig. 3f were calculated by deriving the time from the contractile stroke. The maximum contractile rate and recovery rate of DIYM were 9.4% and −20.8% s^−1^, respectively, while the maximum contractile rate and the recovery rate of the RCYM were only 4.1% and −13.2% s^−1^, respectively (Fig. 3g). With the increasing frequency of the applied square wave potential, the contractile strokes of both two types of yarn muscles decreased (Figs. 3h and S14–S15), which was caused by the limited migration rate of the ions. When the frequency increased to 1 Hz, the contractile stroke of the RCYM decayed to 1.1%, which was 3.6% for DIYM. The power density during contraction increased first and then decreased as the frequency increased since the power density depended on both time and contractile stroke. The maximum power densities of the DIYM and RCYM were 0.37 and 0.16 W g^−1^, respectively. These results further suggested that the dual-ion co-regulation system endowed the yarn muscle with high contractile stroke and fast actuation response.

We further investigated the effects of applied tensions on the contractile stroke and the generated contraction work of the DIYM in Fig. S16 when a square wave at 3 to 5 V at 0.14 Hz with a 72% duty cycle (5 V for 5 s and 3 V for 2 s) was applied. As the applied tension increased, the contractile stroke increased fast and then decreased. The contraction work increased first and then decreased with the increase of the applied tensions, and the maximum contraction work was 1.75 J g^−1^ when the tension was about 13 MPa. This is nearly 44 times that of biological muscle [37], 7.4 times that of CNT/rGO spiral artificial muscle fibers in the inorganic electrolyte (236 J kg^−1^) [21], 66 times that of solvent-driven spiral carbon nanotubes (26.7 J kg^−1^) [39], and 11.6 times that of PDMS/CNT composite helical fibers (150 J kg^−1^) under electrothermal drive [40]. The cyclic stability of the DIYM was evaluated as shown in Fig. S17. The yarn muscle measured by applying a square wave potential at about 0.56 Hz and a tension of 10 MPa showed stable contractile stroke. It only decayed about 0.1%, from 8.6% to 8.5%, after 10,000 continuous rapid contraction cycles. We further characterize the morphological changes of the artificial muscles before and after electrochemical actuating. Figure S18 shows the SEM images of the artificial muscle yarn. After 10,000 continuous rapid contraction cycles, the coils in the yarn are well separated (Fig. S18a) with a diameter of about 83 μm, and the smooth surface of the yarn becomes wrinkled after actuating (Fig. S18b, c). And the strain–stress curve (Fig. S19) shows the failure strain decreases and the breaking strength increases after actuating. This may be due to the large volume expansion and rearrangement of CNTs in the CNT yarn [17].

### Generated Isometric Stress of DIYM

**Fig. 4:**
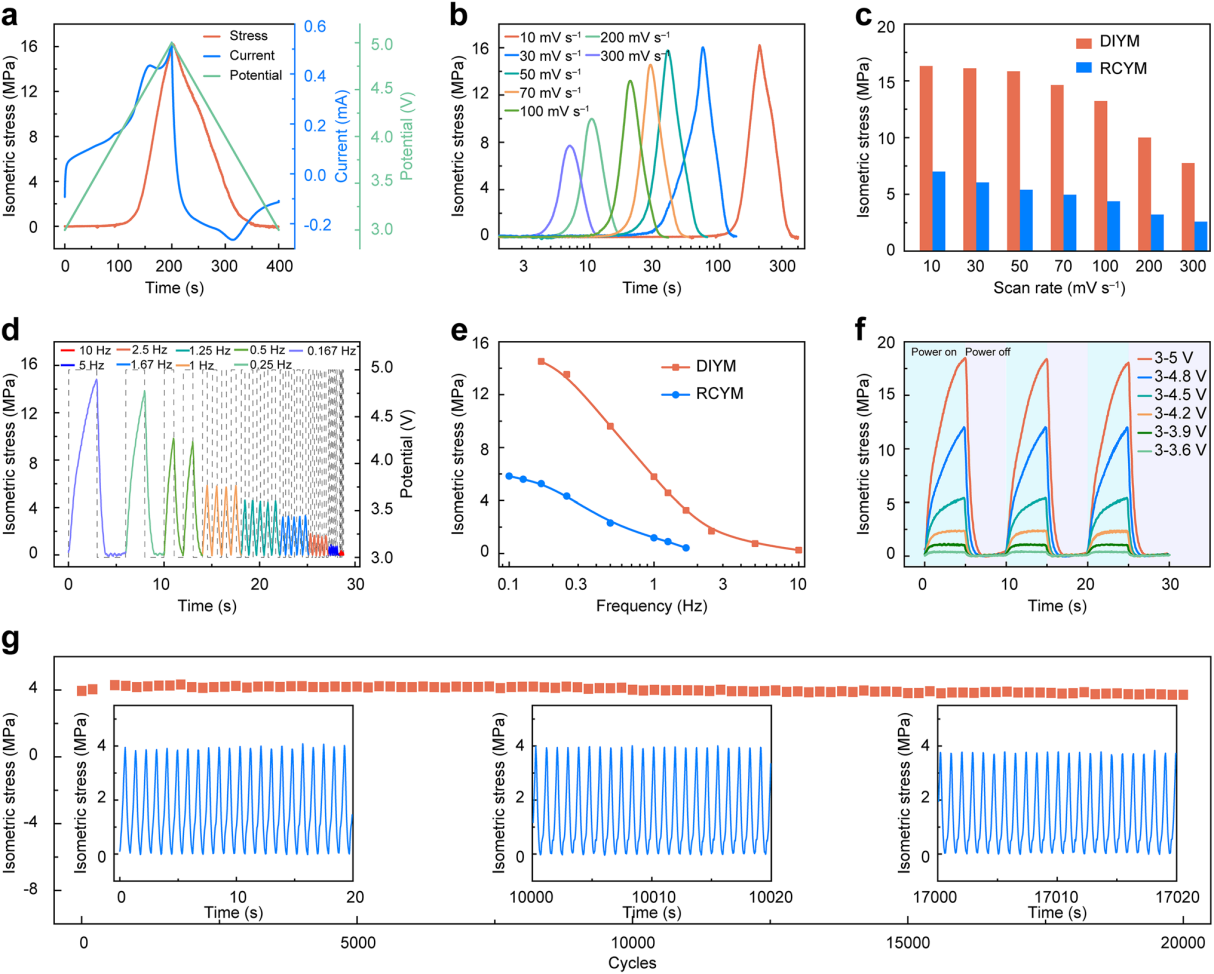
The generated isometric stress of DIYM. **a** Isometric stress, current, and the applied potential versus time during a full CV scan between 3 and 5 V at 10 mV s^−1^ for a coiled yarn muscle bearing 10 MPa applied stress. **b** The isometric stress of the yarn muscle that was charged from 3 to 5 V at different scan rates and the tension loads was 10 MPa. **c** The comparison of the isometric stress of the DIYM and RCYM at different scan rates from 10 to 300 mV s^−1^ and the applied tension loads was 10 MPa. **d** The isometric stress curves under different on/off frequencies when a 3−5 V square wave and tension stress of 10 MPa were applied. **e** The comparison of the isometric stress of the DIYM and RCYM at different frequencies when the 3–5 V and 0.2–2.2 V square wave were applied for DIYM and RCYM, respectively, and tension stress was 10 MPa. **f** The isometric stress versus time during the square wave potential with different potentials at 0.1 Hz for a coiled yarn bearing 10 MPa applied stress. **g** Isometric stress versus cycle number for a yarn muscle when a 1 Hz, 3 to 5 V square wave potential was used

The isometric stress generated by DIYM was investigated, which was an important parameter for evaluating the muscles to support the capabilities of holding objects and pressing [18]. The schematic illustration of the apparatus for isometric stress characterization is shown in Fig. S20. The time dependence of isometric stress and current during a full cycle of CV scan between 3 and 5 V at 10 mV s^−1^ for a coiled CNT yarn under an applied load of 10 MPa was characterized (Fig. 4a). Similar to that of the contractile stroke, the value of isometric stress was relatively small, while the current remained positive and gradually increased during the first 120 s. And then the isometric stress quickly increased from 0.47 MPa at 4.2 V to 16 MPa at 5 V, which suggested that the insertion of ${\text{PF}}_{6}^{ - }$ ions at this voltage range caused a large contractile stroke. During the discharging process, the isometric stress decreased to 0. With increasing the scan rates at the CV scan between 3 and 5 V, the isometric stress remained stable at the scan rate below 70 mV s^−1^ and gradually decreased when the scan rates were over 70 mV s^−1^ (Fig. 4b), while compared with DIYM, the isometric stress of RCYM significantly decreased with increasing scanning rates from 10 to 300 mV s^−1^ (Fig. S21). As shown in Fig. 4c, the isometric stresses of DIYM were 16.3, 16.1, 15.8, and 14.6 MPa for scan rates of 10, 30, 50, and 70 mV s^−1^, respectively, and the isometric stress only attenuated by about 10% when the scan rate was increased by a factor of 7. And the isometric stresses of the RCYM were only 7, 6, 5.4, and 4.9 MPa at the corresponding scan rates, respectively. When the scan rate was increased to 300 mV s^−1^, the isometric stresses of DIYM were still 7.8 MPa, which was about 3 times that of the RCYM at the same scan rate (2.6 MPa). The comparison of the main actuation performance parameters (including isometric stress, contractile stroke, contractile rate, power density, and energy conversion efficiency) between DIYM and RCYM shows that the shortened ion migration pathways by the dual-ion co-regulation system enhanced main actuation performances of DIYM (Fig. S22).

The isometric stress of the DIYM at different on/off frequencies of the applied potential is shown in Fig. 4d. When the 5 V potential was applied for 3 s, the isometric stress increased to a maximum value of 14.5 MPa, and then the isometric stress gradually decreased to 0 when the potential was switched to 3 V. The isometric stress generated by the two types of yarn muscle decreased as the frequency increased (Figs. 4d and S23). Increasing the frequency and the scan rate led to a decrease in the isometric stress confirming that the migration rate of the ions determined the high-frequency response performance of artificial muscles. The comparison of the isometric stresses generated by the two types of artificial yarn muscle at different frequencies showed that the DIYM generated greater isometric stress at high frequency (Fig. 4e). When the frequency increased to 1.67 Hz, the isometric stress of RCYM decreased to approximately 0.4 MPa, while that of DIYM was 3.3 MPa (about 8 times than that of RCYM). And the isometric stress generated by the DIYM remained at 0.3 MPa even at a frequency of 10 Hz, which is comparable to biological muscle. The isometric stress rate is shown in Fig. S24. The maximum isometric stress rate of the DIYM is 11.6 MPa s^−1^ at 1 Hz, which is about 5 times that of the RCYM (about 2.4 MPa s^−1^ at 1 Hz). The higher isometric stress at high frequency and the higher isometric stress rate of the DIYM are mainly due to the fast ion migration in this system.

Then, we investigated the isometric stress of the DIYM under different potentials using a square wave at 0.1 Hz (Figs. 4f and S25). The isometric stress increased as the potential increased (the slope of the curve in Fig. S25). The curves exhibited higher increase rates at the square wave potential of 3–4.5, 3–4.8 and 3–5 V, which is consistent with no obvious generated stress during the first 120 s in Fig. 4a. This is due to the intercalation reaction that produced large isometric stress occurs at a potential above 4.2 V. At the potential of 3−3.6, 3–3.9, and 3–4.2 V, the isometric stress increased first and then reached a platform, while no platforms were observed at potentials of 3–4.5, 3–4.8, and 3–5 V, which can be explained by ion adsorption with a faster reaction kinetics than the intercalation reaction. The maximum value was about 18.4 MPa when the 5 V potential was applied for 5 s, which is about 61 times that of skeletal muscles (0.35 MPa) [37] and much higher than other reported electrochemical artificial muscles (Fig. S26), such as Bucky gel (0.1 MPa) [41], SWNT sheet (0.75 MPa) [38], CNT-rGO yarn (4 MPa) [21], ITAP CNT yarn (4.2 MPa) [17], CNT@PVDF yarn (10.8 MPa) [18], and CNT yarn (14.6 MPa) [25].

Finally, the cycling stability of the isometric stress was evaluated by applying a 3 to 5 V square wave at 1 Hz with a 50% duty cycle as shown in Fig. 4g. The isometric stress decreased from 3.9 to 3.7 MPa after 20,000 continuous rapid contraction cycles. This indicates the good cyclic stability of the generated isometric stress of artificial yarn muscle by faradaic ${\text{PF}}_{6}^{ - }$ insertion and de-insertion.

## Conclusions

In summary, the electrochemical artificial yarn muscles driven in a dual-ion co-regulation system have exhibited high contractile stroke, large isometric stress, and fast actuation response while maintaining the catch state. The high performance is enabled by the dual-ion co-regulation system, which shortens ion migration pathways by providing two reaction channels where the anion and cation react with the anode and cathode, respectively. This reaction mechanism allows the DIYM to show 3, 2, and 8 times the contractile stroke, the maximum contractile rate, and the high-frequency isometric stress of RCYM, respectively. We believe that the dual-ion coordinated driving mechanism can also provide a promising strategy for the development of artificial muscles with high-performance and low-energy consumption.

### Supplementary Information

Below is the link to the electronic supplementary material.

Supplementary file1 (MP4 5276 KB)

Supplementary file2 (PDF 1610 KB)
