# Supplementary material for: Dual-Ion Co-Regulation System Enabling High-Performance Electrochemical Artificial Yarn Muscles with Energy-Free Catch States
Source: Nanomicro Lett. 2023 Jun 29;15:162. doi: 10.1007/s40820-023-01133-2 (PMC10310689; doi:10.1007/s40820-023-01133-2)
Supplement: Supplementary file 2 — Supplementary file2 (PDF 1610 KB) [file 40820_2023_1133_MOESM2_ESM.pdf]

Supporting Information for

Dual-Ion Co-Regulation System Enabling High-Performance Electrochemical Artificial Yarn Muscles with Energy-Free Catch States

Ming Ren[1, 2], Lizhong Dong[1, 2], Xiaobo Wang[1, 2], Yuxin Li[1, 2], Yueran Zhao[1], Bo Cui[1, 2], Guang Yang[1, 2], Wei Li[1], Xiaojie Yuan[1], Tao Zhou[3], Panpan Xu[1], Xiaona Wang[1], Jiangtao Di[1, 2, 3, *], and Qingwen Li[1, 2, 3, *]

[1]Key Laboratory of Multifunctional Nanomaterials and Smart Systems, Advanced Materials Division, Suzhou Institute of Nano-Tech and Nano-Bionics, Chinese Academy of Sciences, Suzhou 215123, P. R. China

[2]School of Nano-Technology and Nano-Bionics, University of Science and Technology of China, Hefei 230026, P. R. China

[3]Division of Nanomaterials and Jiangxi Key Lab of Carbonene Materials, Jiangxi Institute of Nanotechnology, Nanchang 330200, P. R. China

*Corresponding authors. E-mail: jtdi2009@sinano.ac.cn (Jiangtao Di); qwli2007@sinano.ac.cn (Qingwen Li)

Supplementary Figures and Tables

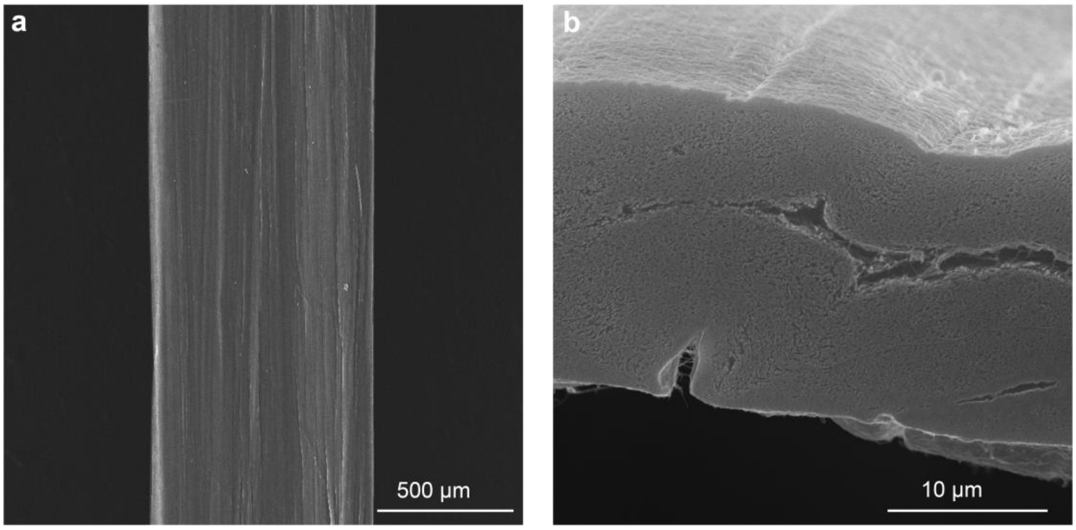

Fig. S1 SEM images of the CNT ribbon. (**a**) The morphology of the CNT ribbons. (**b**) The cross-section of the CNT ribbon

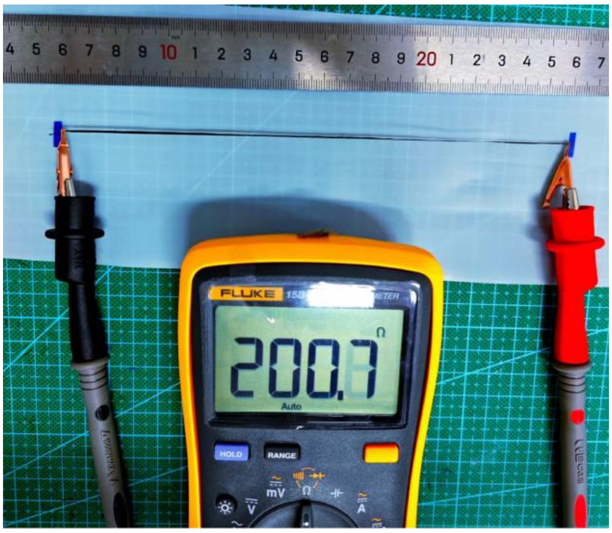

Fig. S2 The optical images of the resistance test of the CNT ribbon. The 20 cm-CNT ribbon has a resistance of 200.7 Ω. The line resistance of the CNT ribbon is about 10 $\Omega\ cm^{-1}$

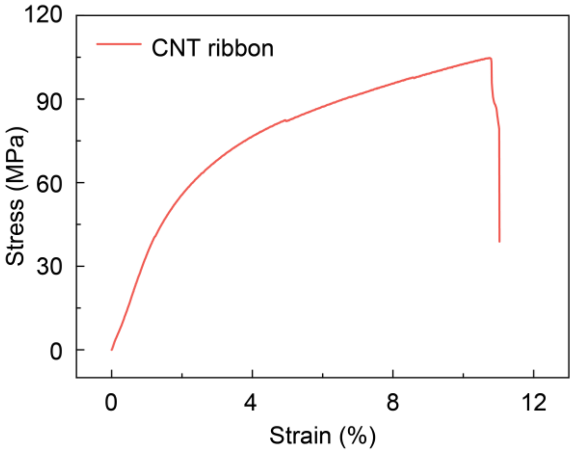

Fig. S3 The strain-stress curve of CNT ribbon. The failure strain is 10.8% and the breaking strength is 104.5 MPa

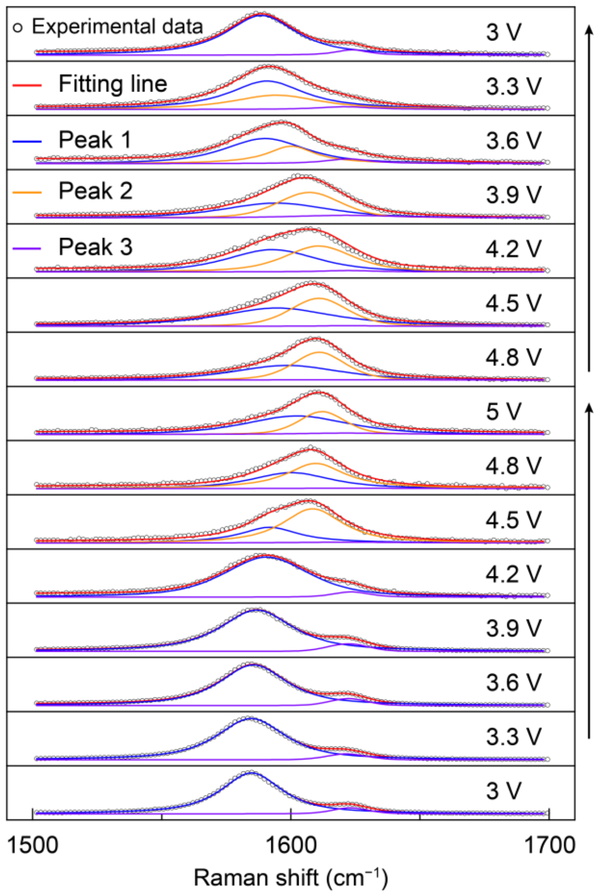

Fig. S4 The ex-situ Raman peak shift of CNT yarn at different charged and discharged states by using CV scan at 10 mV s^{-1}. The ex-situ Raman spectroscopy was performed by using a HeNe laser with a wavelength of 532 nm. The G peak can be fitted into three peaks: peaks 1, 2, and 3 are assigned to the G peak after splitting, the appeared new peak, and the D′ peak, respectively

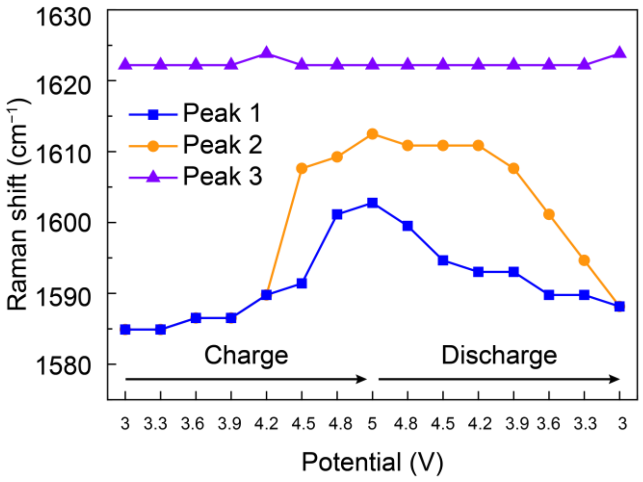

Fig. S5 The Raman shifts of the fitted peaks in Fig. S2. The G peak (peak 1) blue-shifted slightly from 1585 cm^{-1} to 1590 cm^{-1} with increasing the potential from 3 V to 4.2 V. As the coiled CNT yarn was charged to 5 V, the split new peak (peak 2) and the G peak after splitting blue-shifted to the maximum of 1612 and 1603 cm^{-1},

respectively. The two peaks gradually combined into one peak for the G band at 1588 cm^{-1} during the potential back to 3 V. The D′ peak (peak 3) was stable during the charge and discharge process.

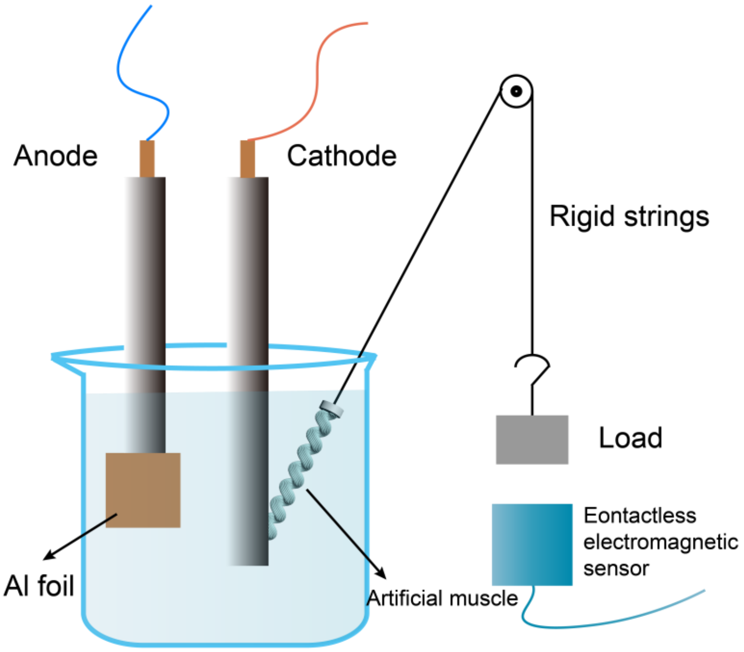

Fig. S6 The schematic illustration of the apparatus for contractile stroke characterization

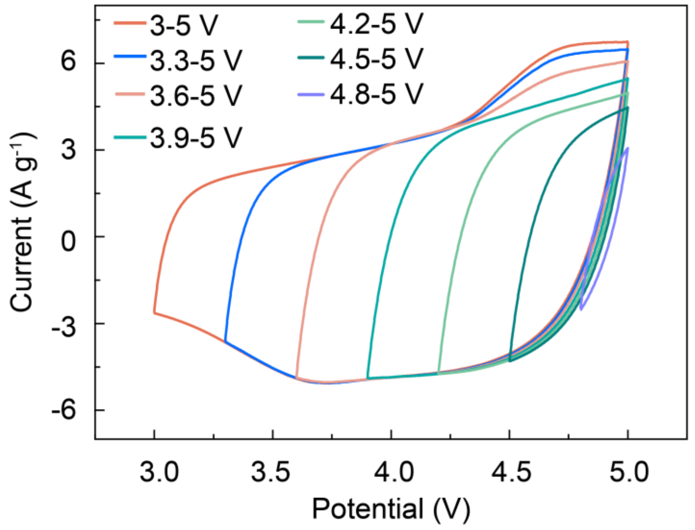

Fig. S7 CV curves of DIYM using CV scan from different onset potentials to 5 V at 50 mV s^{-1} and the applied load was 10 MPa

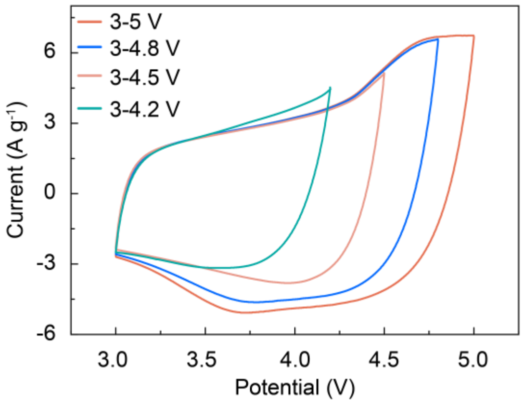

Fig. S8 CV of DIYM using CV scan from 3 V to different end potentials at 50 mV s^{-1} and the applied load was 10 MPa

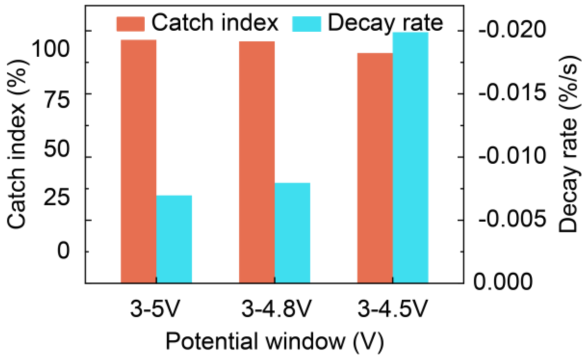

Fig. S9 The decay rates of the catch index of the DIYM when the power was off for 500 s. The artificial muscle yarn was charged from 3 V at a scan rate of 50 mV s^{-1} to 4.5, 4.8, and 5 V, respectively, and then the power is off. The catch index at different end potentials of 4.5, 4.8, and 5 V were 91.2%, 95.8%, and 96.4%, respectively, and the decay rates of the catch index were 0.02, 0.008, and 0.007 %·s^{-1}, respectively

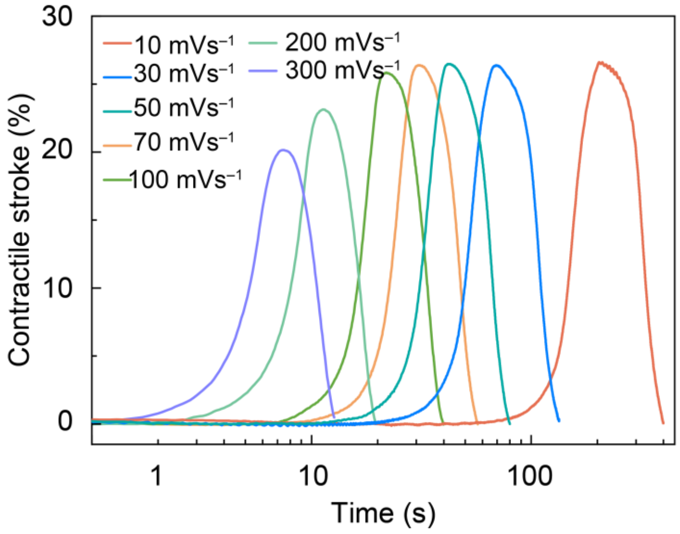

Fig. S10 The curves of contractile stroke of the DIYM versus time at different scan rates during CV measurements from 3 V to 5 V at the applied load of 10 MPa

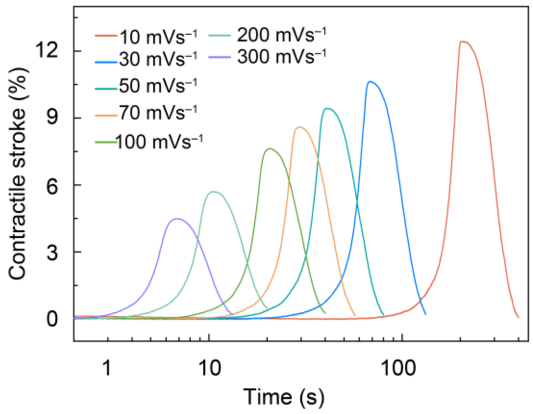

Fig. S11 The curves of contractile stroke of the RCYM versus time at different scan rates during CV measurements from 3 V to 5 V at the applied load of 10 MPa

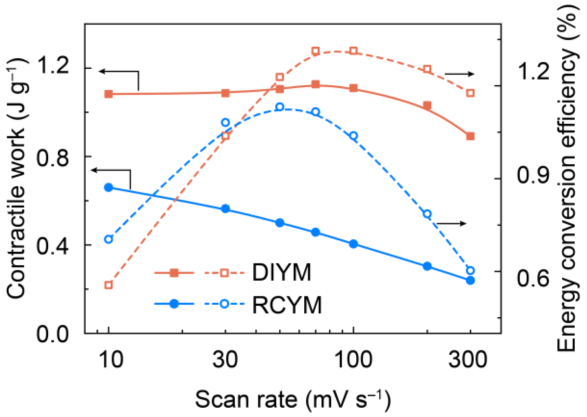

Fig. S12 The maximum contraction work and energy conversion efficiency of DIYM during CV scan from 3 V to 5 V and the RCYM during CV scan from 0.2 V to 2.2 V at different scan rates and the applied load is 10 MPa

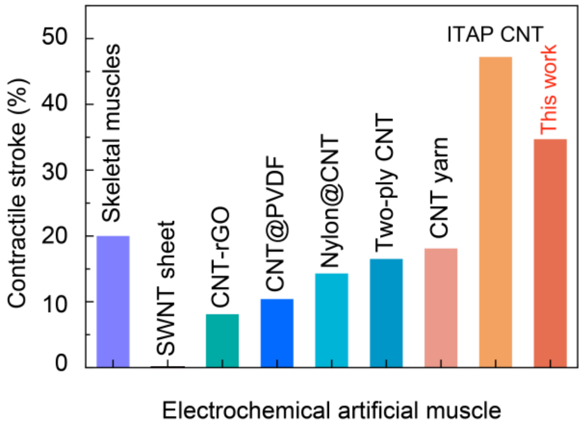

Fig. S13 Comparison of the contractile stroke of DIYM with mammalian skeletal muscles (20%), [1] electrochemical muscles (SWNT sheet (0.1%), [2] CNT-rGO yarn (8.1%), [3] CNT@PVDF yarn (10.4%), [4] Nylon/CNT (14.3%), [5] Two-ply CNT yarn (16.5% MPa), [10] CNT yarn (18.1%), [6] and ITAP CNT yarn (47.2%) [7]

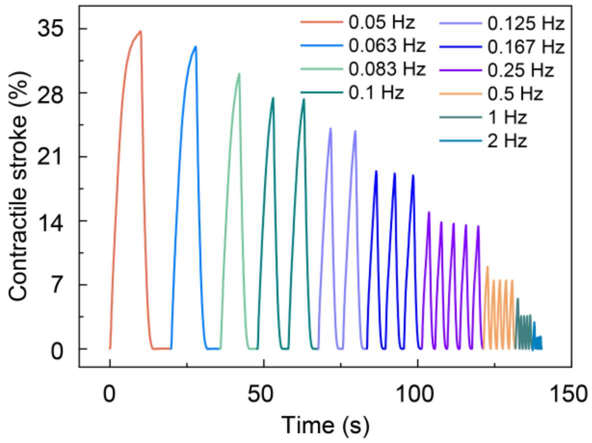

Fig. S14 The curves of contractile stroke of the DIYM versus time at different on/off frequencies when a 3 to 5 V square wave with a 50% duty cycle and tension stress of 10 MPa were applied

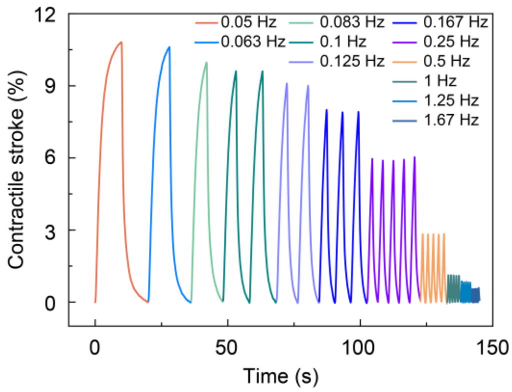

Fig. S15 The curves of contractile stroke of the RCYM versus time at different on/off frequencies when a 3 to 5 V square wave with a 50% duty cycle and tension stress of 10 MPa were applied

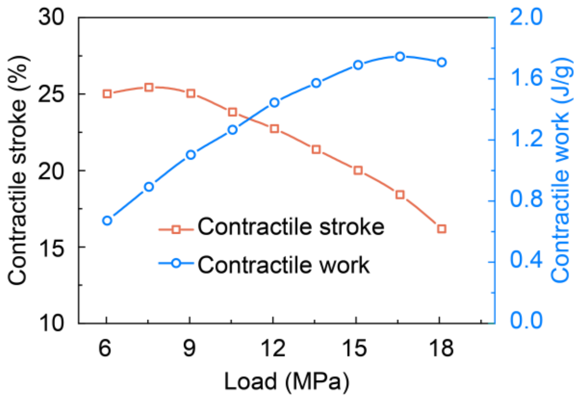

Fig. S16 The effects of applied tensions on the contractile stroke and the generated contraction work of the DIYM when a 3 to 5 V square wave at 0.14 Hz with a 72% duty cycle (5 V for 5 s and 3 V for 2 s) were applied

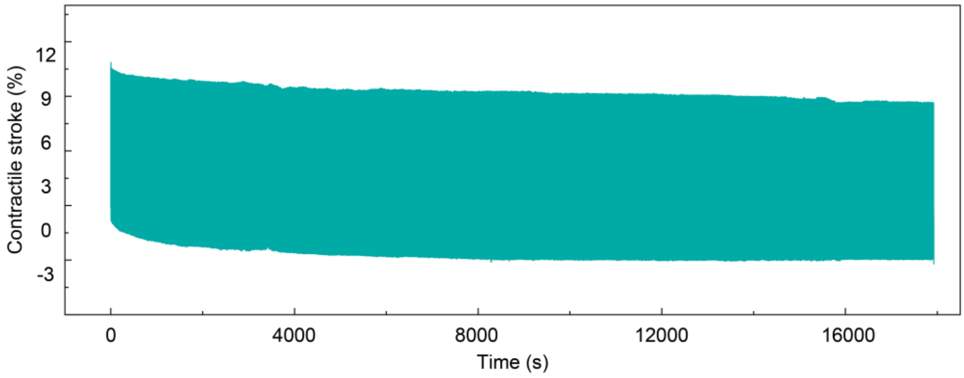

Fig. S17 The cyclic stability of the DIYM. The potential of 5 V was held for about 0.9 s, then switched to 3 V for about 0.9 s, and the response frequency was 0.24 Hz, while a pre-tensile tension of 10 MPa was applied.

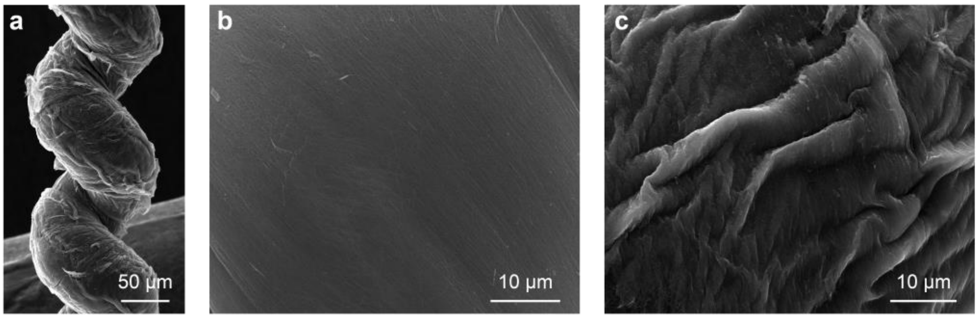

Fig. S18 The cyclic stability of the DIYM. The potential of 5 V was held for about 0.9 s, then switched to 3 V for about 0.9 s, and the response frequency was 0.24 Hz, while a pre-tensile tension of 10 MPa was applied.

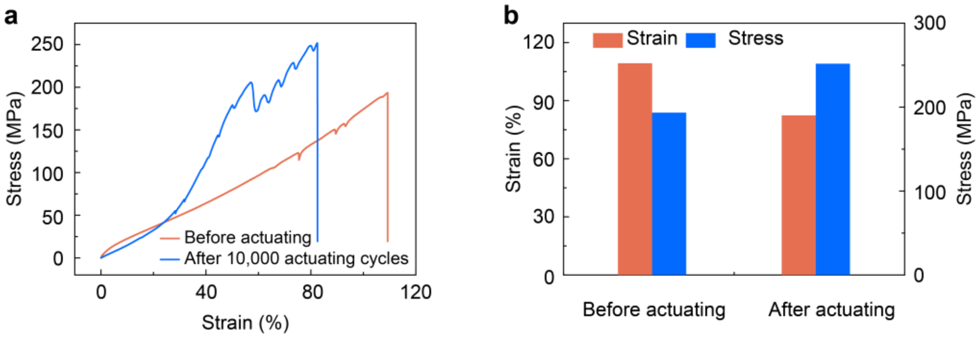

Fig. S19 The mechanical properties of the CNT yarns before and after 10,000 continuous rapid contraction cycles. (a) The stress-strain curves. (b) The comparison of the failure strain and the breaking strength between the artificial muscle before and after actuating. The failure strain and the breaking strength of the CNT yarn before the test are 109.3% and 193.4 MPa, respectively. And, after 10,000 continuous rapid contraction cycles, the failure strain and the breaking strength of the CNT yarn before the test are 82.4% and 251.7 MPa, respectively.

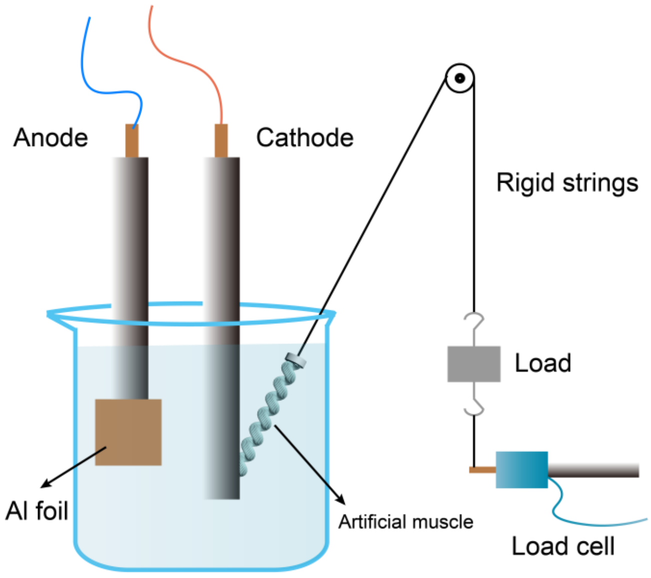

Fig. S20 The schematic illustration of the apparatus for isometric stress characterization

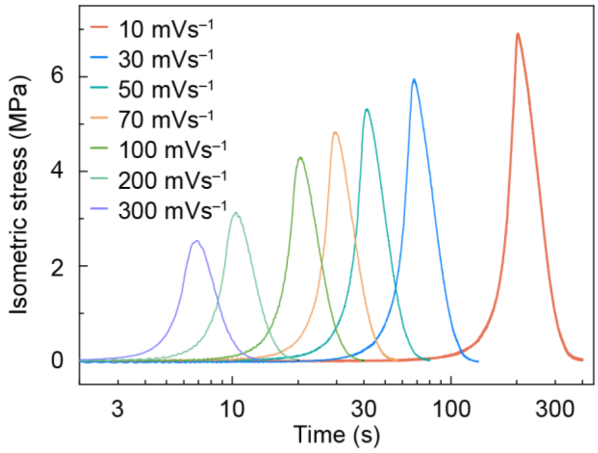

Fig. S21 The curves of isometric stress of the RCYM versus time at different scan rates during CV measurements from 3 V to 5 V at the applied load of 10 MPa

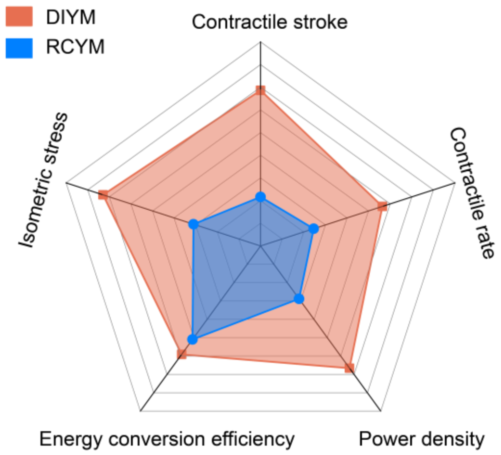

Fig. S22 The comparison of the actuation performances of the DIYM and RCYM

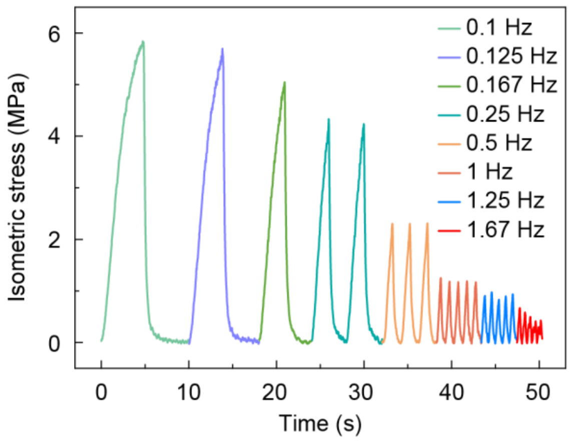

Fig. S23 The curves of isometric stress of the RCYM versus time at different on/off frequencies when a 3 to 5 V square wave with a 50% duty cycle and tension stress of 10 MPa were applied

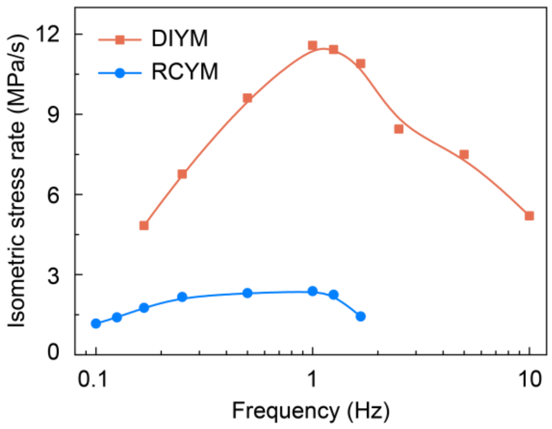

Fig. S24 The comparison of the isometric stress rate of the DIYM and RCYM at different frequencies when the 3−5 V and 0.2−2.2 V square wave were applied for DIYM and RCYM, respectively, and tension stress was 10 MPa

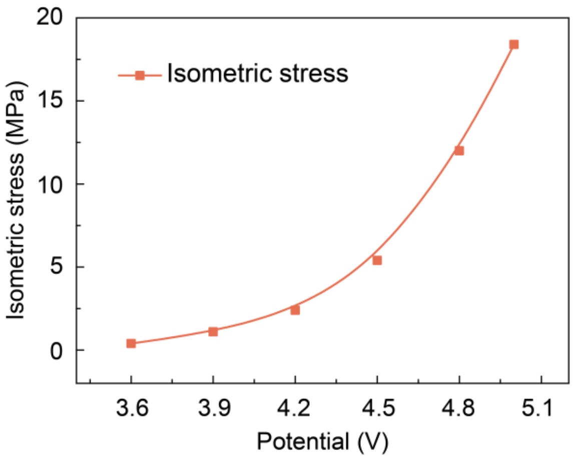

Fig. S25 The isometric stress of the DIYM under square waves with different potentials at 0.1 Hz. The potential for the returning process of yarn muscle was 3 V and the tension stress of was10 MPa

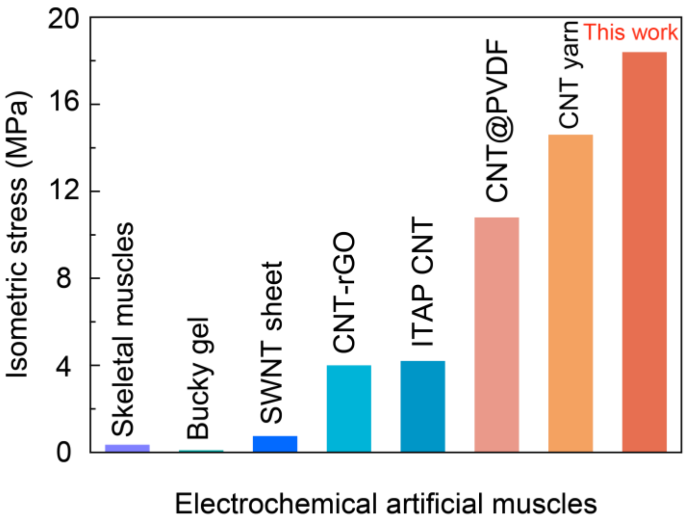

Fig. S26 Comparison of the generated isometric stress with mammalian skeletal muscles (0.35 MPa), [S1] electrochemical muscles (Bucky gel (0.1 MPa), [S8] SWNT sheet (0.75 MPa), [S2] CNT-rGO yarn (4 MPa), [S3] ITAP CNT yarn (4.2 MPa), [S7] CNT@PVDF yarn (10.8 MPa), [S4] and CNT yarn (14.6 MPa) [S6]

Supplementary References

[S1] T. Mirfakhrai, J.D.W. Madden, R.H. Baughman. Polymer artificial muscles. Mater Today **10**(4), 30-38 (2007). https://doi.org/10.1016/S1369-7021(07)70048-2

[S2] R. H. Baughman, C. Cui, A. A. Zakhidov, Z. Iqbal, J. N. Barisci et al., Carbon nanotube actuators. Science **284**(5418), 1340-1344 (1999). https://doi.org/10.1126/science.284.5418.1340

[S3] J. Qiao, J. Di, S. Zhou, K. Jin, S. Zeng et al., Large-stroke electrochemical carbon nanotube/graphene hybrid yarn muscles. Small **14**(38), 1801883 (2018). https://doi.org/10.1002/smll.201801883

[S4] M. Ren, J. Qiao, Y. Wang, K. Wu, L. Dong et al., Strong and robust electrochemical artificial muscles by ionic-liquid-in-nanofiber-sheathed carbon nanotube yarns. Small **17**(5), e2006181 (2021). https://doi.org/10.1002/smll.202006181

[S5] J. Mu, M. Jung de Andrade, S. Fang, X. Wang, E. Gao et al., Sheath-run artificial muscles. Science **365**(6449), 150-155 (2019). https://doi.org/10.1126/science.aaw2403

[S6] M. Ren, P. Xu, Y. Zhou, Y. Wang, L. Dong et al., Stepwise artificial yarn muscles with energy-free catch states driven by aluminum-ion insertion. ACS Nano **16**(10), 15850-15861 (2022). https://doi.org/10.1021/acsnano.2c05586

[S7] Y. L. Wang, Y. R. Zhao, M. Ren, Y. R. Zhou, L. Z. Dong et al., Artificial muscle fascicles integrated with high-performance actuation properties and energy-

storage function. Nano Energy **102**, 107609 (2022). https://doi.org/10.1016/j.nanoen.2022.107609

[S8] T. Fukushima, K. Asaka, A. Kosaka, T. Aida. Fully plastic actuator through layer-by-layer casting with ionic-liquid-based bucky gel. Angew. Chem. Int. Ed. **44**(16), 2410-2413 (2005). https://doi.org/10.1002/anie.200462318
